# Pathogenic Escherichia coli Hijacks GTPase-Activated p21-Activated Kinase for Actin Pedestal Formation

**DOI:** 10.1128/mBio.01876-19

**Published:** 2019-08-20

**Authors:** Vikash Singh, Anthony Davidson, Peter J. Hume, Vassilis Koronakis

**Affiliations:** a Department of Pathology, University of Cambridge, Cambridge, United Kingdom; Harvard Medical School; Imperial College London; University of Connecticut

**Keywords:** actin, EPEC, EspG, GTPases, p21-activated kinases

## Abstract

Enteropathogenic Escherichia coli and enterohemorrhagic E. coli (EPEC and EHEC, respectively) are extracellular pathogens that reorganize the host cell cytoskeleton to form “actin pedestals” beneath the tightly adherent bacteria, a critical step in pathogenesis. EPEC and EHEC inject effector proteins that manipulate host cell signaling cascades to trigger pedestal assembly. One such effector, EspG, has been reported to bind and activate p21-activated kinase (PAK), a key cytoskeletal regulator, but the function of this interaction and whether it impacts pedestal assembly are unknown. Here, we demonstrate that deletion of *espG* significantly impairs pedestal formation and attachment by both EPEC and EHEC. This role of EspG is shown to be dependent on its interaction with PAK. Unexpectedly, EspG was able to subvert PAK only in the presence of Rho family small GTPases, which function to both concentrate PAK at the membrane and stimulate PAK activation. Our findings reveal a novel mechanism by which EspG hijacks PAK and sustains its active state to drive bacterial attachment to host cells.

## Abstract (precis)

Enteropathogenic E. coli and enterohemorrhagic E. coli (EPEC and EHEC, respectively) remain a significant global health problem. Both EPEC and EHEC initiate infection by attaching to cells in the host intestine, triggering the formation of actin-rich “pedestal” structures directly beneath the adherent pathogen. These bacteria inject their own receptor into host cells, which upon binding to a protein on the pathogen surface triggers pedestal formation. Multiple other proteins are also delivered into the cells of the host intestine, but how they contribute to disease is often less clear. Here, we show how one of these injected proteins, EspG, hijacks a host signaling pathway for pedestal production. This provides new insights into this essential early stage in EPEC and EHEC disease.

## INTRODUCTION

Infectious diarrheal diseases are a significant public health burden worldwide and remain a leading cause of infant mortality (1). Enteropathogenic Escherichia coli (EPEC) causes diarrhea in children, especially in the developing world (2), while enterohemorrhagic E. coli (EHEC) is associated with outbreaks of bloody diarrhea in the developed world, sometimes leading to life-threatening complications, such as hemolytic uremic syndrome (3). EPEC and EHEC tightly adhere to intestinal epithelial cells and cause morphological changes leading to the loss of brush border microvilli, forming characteristic attaching and effacing (A/E) lesions (4). Extensive reorganization of the cytoskeleton beneath the adherent pathogens leads to the formation of actin “pedestals,” which strengthen the attachment of the bacteria to the host epithelium, a crucial step in pathogenesis (5).

EPEC and EHEC trigger pedestal formation by injecting their own receptor (translocated intimin receptor [Tir]) into target cells (6), although the precise pathways downstream of Tir differ. Upon binding to the bacterial surface protein intimin and consequent clustering in the host cell plasma membrane, EPEC Tir is phosphorylated by host kinases, triggering the recruitment of host adaptor proteins, activation of neuronal Wiskott-Aldrich syndrome protein (N-WASP), and consequent ARP2/3-dependent actin assembly (7–10). EHEC Tir does not rely on phosphorylation but binds the host proteins IRTKS and IRSp53 to recruit the EHEC effector EspF-like protein encoded on phophage U (EspF_U_), which multimerizes N-WASP to promote Arp2/3-driven actin assembly (11, 12). While Tir is central to actin pedestal formation in cultured cells, this pathway alone is not sufficient to allow A/E lesion formation in *in vitro* organ culture models (13). Both EPEC and EHEC also inject myriad other effector proteins that coordinately manipulate host cell signaling to promote colonization and pathogenesis (14).

EspG is one such effector protein (15), delivered by both EPEC and EHEC (EPEC also encodes a second EspG homologue, EspG2, which is 42% identical and 62% similar to EspG [16]). Functions reported for EspG include disruption of microtubule networks, the loss of epithelial barrier function, a decrease in transepithelial resistance, the arrest of vesicle traffic, and blocking of recycling of vesicle cargo to the cell surface (17–20). Several biochemical activities are thought to be responsible for these various cellular functions. EspG can bind to host Arf GTPases, turn off Rab GTPases by acting as a GTPase-activating protein (GAP), and bind and activate p21-activated kinases (PAKs) (21–23). Arf and PAK are important regulators of the host actin cytoskeleton (24, 25). Indeed, we recently reported that by binding to Arf, EspG can block the phagocytosis of EPEC by macrophages (26). We therefore assessed whether EspG has an additional role in the actin remodeling underlying pedestal formation and the consequent adhesion of EPEC/EHEC to target host cells.

## RESULTS

### EspG promotes pedestal formation and strong bacterial attachment.

**FIG 1:**
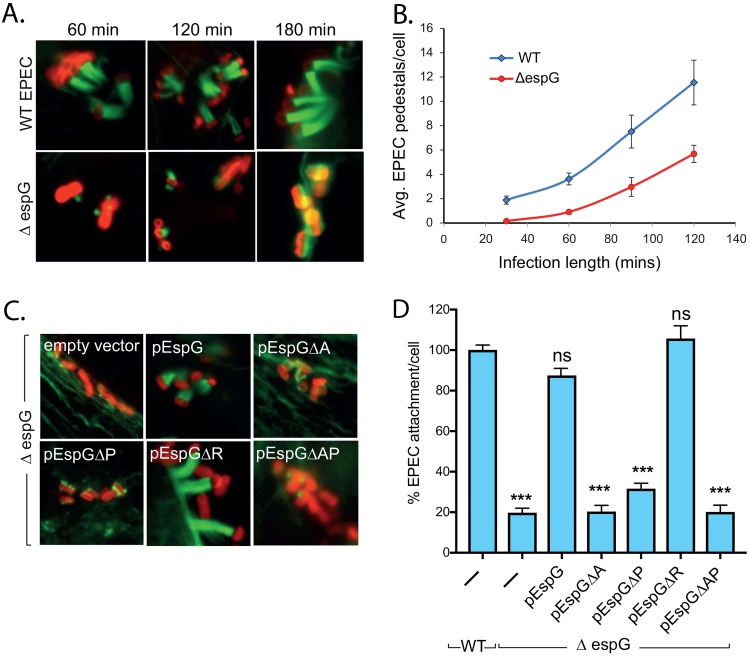
EspG promotes bacterial attachment and pedestal formation. (A) Fluorescence microscopy images of actin pedestals formed on Hap1 cells by WT and Δ*espG* EPEC bacteria at the times indicated. Actin (green) is stained with Alexa Fluor 488-phalloidin, and bacteria (red) are stained with anti-intimin antibody. Scale bar, 1 μm. (B) Quantification of the number of pedestals formed by WT and Δ*espG* EPEC bacteria at the indicated time points. Each data point represents the average of results from 3 separate experiments (300 to 500 cells for each experiment). Error bars indicate standard deviations (SD). (C) Fluorescence microscopy images of actin pedestals formed on Hap1 cells by WT EPEC, Δ*espG* EPEC, or Δ*espG* EPEC transformed with plasmids encoding WT EspG or an EspG derivative defective in binding to either Arf (EspGΔA), PAK (EspGΔP), Rab (EspGΔR), or both Arf and PAK (EspGΔAP). Actin (green) is stained with Alexa Fluor 488-phalloidin, and bacteria (red) were stained with anti-intimin antibody. Scale bar, 1 μm. (D) Quantification of the attachment of strains from panel C to WT Hap1 cells, relative to that of WT EPEC (there are typically 6 to 7 WT EPEC bacteria per cell). Each bar represents the average of results from 3 separate experiments (300 to 500 cells for each experiment). Error bars indicate SD. ***, *P* < 0.001; ns, not significant (by one-way analysis of variance [ANOVA] followed by a *post hoc* Dunnett comparison) relative to WT EPEC attachment.

To establish the contribution of EspG to pedestal formation, Hap1 cells were infected with either wild-type (WT) EPEC or an *espG1 espG2* double-knockout strain (herein the Δ*espG* mutant), and actin pedestal formation was examined by fluorescence microscopy (Fig. 1A). Pedestals induced by Δ*espG* EPEC were much shorter and contained less actin than those formed by WT EPEC. The same phenotype was also seen in Caco-2 cells, mouse embryonic fibroblasts (MEFs), and HeLa cells (see Fig. S1A in the supplemental material). Pedestal formation by Δ*espG* EPEC did eventually occur but was slow, with significantly fewer pedestals than those of the WT at all time points examined (Fig. 1B). EspG was also required for the correct generation of pedestals by EHEC (Fig. S1B), even though EHEC uses a different mechanism to trigger actin rearrangements. Importantly, pedestal formation in Δ*espG* EPEC could be fully recovered by expressing WT EspG (Fig. 1C). An EspG mutant deficient in binding Rab GTPases (EspGΔR mutant) could also restore pedestals and, in fact, promoted slightly longer pedestals than those of the WT (Fig. S1C). However, mutant EspGs unable to interact with Arf, PAK, or both Arf and PAK (EspGΔA, EspGΔP, and EspGΔAP, respectively) could not recover pedestal formation (Fig. 1C and Fig. S1C).

10.1128/mBio.01876-19.1 FIG S1 (A) Fluorescence microscopy images of pedestals formed by WT or Δ*espG* EPEC on Caco-2 cells, MEFs, or HeLa cells, as indicated. Bacteria were visualized using an anti-intimin antibody (red), while Alexa Fluor 488-phalloidin (green) was used to stain actin. Scale bar, 5 μm. (B) Fluorescence microscopy images of pedestals formed by WT or Δ*espG* EHEC on WT Hap1 cells. Bacteria were visualized using an anti-intimin antibody (red), while Alexa Fluor 488-phalloidin (green) was used to stain actin. Scale bar, 5 μm. (C) Average length of actin pedestals formed during the experiment described in the legend of Fig. 1C. ***, *P* < 0.001; **, *P* < 0.01; ns, not significant (one-way ANOVA followed by a *post hoc* Dunnett comparison) relative to those formed by WT EPEC. (D) Representative fluorescence microscopy image showing the attachment of WT and Δ*espG* EPEC bacteria to WT Hap1 cells after they were washed with PBS and briefly with glycine (pH 2) postinfection. Bacteria were stained using an anti-intimin antibody (red), and Alexa Fluor 488-phallodin (green) was used to stain actin. Scale bar, 10 μm. (E) Quantification of the attachment of Δ*espG* EHEC bacteria to Hap1 cells, relative to that of WT EHEC (typically 5 to 6 per cell). Each bar represents the average of results from 3 separate experiments (300 to 500 cells for each experiment). Error bars represent SD. ***, *P* < 0.001 (Student’s t test). (F and G) Quantification of the attachment of Δ*espG* EPEC bacteria to Hap1 cells and MEFs, relative to that of WT EPEC at 90 min (F) and 180 min (G) postinfection. ***, *P* < 0.001 (Student’s *t* test) relative to the attachment of WT EPEC. Download FIG S1, PDF file, 0.1 MB. Copyright © 2019 Singh et al. 2019 Singh et al. This content is distributed under the terms of the Creative Commons Attribution 4.0 International license.

To determine the functional consequences of defective pedestal generation, Hap1 cells were infected for 90 min and subsequently subjected to a brief acidic wash to remove weakly adherent bacteria. For both EPEC and EHEC, significantly fewer Δ*espG* than WT bacteria were left attached to cells (Fig. 1D, S, and D and Fig. S1E). A similar defect in attachment was seen after 180 min and also in MEFs (Fig. S1F and G). As seen above for pedestal number, the defect in attachment could be restored by expressing either EspG or EspGΔR but not by expressing EspGΔA, EspGΔP, or EspGΔAP (Fig. 1D). Therefore, EspG has an important role in promoting pedestal formation and consequent attachment of EPEC to host cells, and this activity requires the binding of EspG to both Arf and PAK but not Rabs.

### PAK is required for efficient pedestal formation.

**FIG 2:**
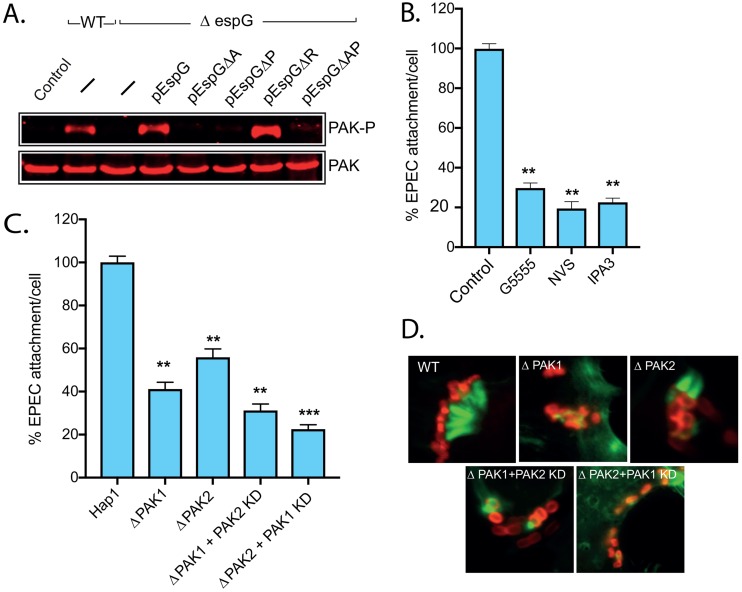
Active PAK is essential for bacterial attachment and pedestal formation. (A) Immunoblot of the level of total PAK and active (i.e., phosphorylated on serine 144) PAK (PAK-P) in Hap1 cells infected with the indicated strains of EPEC. (B) Attachment of WT EPEC to Hap1 cells pretreated with DMSO (control) or the PAK inhibitor G5555, NVS-PAK-1-1, or IPA3. Values are relative to attachment to control Hap1 cells (typically 6 to 7 per cell). Each bar represents the average of results from 3 separate experiments (300 to 500 cells for each experiment). Error bars indicate SD. (C) Attachment of WT EPEC to ΔPAK1 and ΔPAK2 Hap1 cells, with and without additional knockdown of the remaining PAK isoform using siRNA. Values are relative to attachment in WT Hap1 cells (there are typically 6 to 7 per cell). Each bar represents the average of results from 3 separate experiments (300 to 500 cells for each experiment). Error bars indicate SD. (D) Fluorescence microscopy images of EPEC pedestals on cells infected as described for panel C. Actin is stained with Alexa Fluor 488-phalloidin (green), and bacteria are stained with an anti-intimin antibody (red). Scale bar, 1 μm. ***, *P* < 0.001; **, *P* < 0.01 (one-way ANOVA followed by a *post hoc* Dunnett comparison) relative to control (B) or WT Hap1 (C) cells.

Because EspG has been reported to bind and activate group I PAKs (PAK1, -2, and -3) *in vitro* (21, 22) and the results above suggest that the interaction between EspG and PAK is of importance in pedestal formation and bacterial attachment, we tested the ability of EspG to activate PAK during infection. Hap1 cells were infected for 90 min, and then PAK activation status was determined by Western blotting (Fig. 2A). In resting cells, PAK is auto-inhibited and, upon activation, adopts an “open” conformation, stabilized by the autophosphorylation of various residues (25). Autophosphorylation is therefore a readout for the formation of the open active state. WT EPEC infection resulted in a significant increase in the autophosphorylation of serines 141 and 144 of PAK1 and -2, respectively, whereas the Δ*espG* strain triggered no increase relative to the level in uninfected control cells. Similar results were seen for EHEC (Fig. S2A). As seen above for pedestal formation, PAK activation triggered by the Δ*espG* strain could be restored to the level induced by WT EPEC by expressing EspG or EspGΔR but not by expressing EspGΔA, EspGΔP, or EspGΔAP (Fig. 2A).

10.1128/mBio.01876-19.2 FIG S2 (A) Immunoblot showing the level of active (PAK-P) and total (PAK) PAK in uninfected Hap1 cells or those infected with either WT or Δ*espG* EHEC bacteria. (B) Immunoblot depicting active (PAK-P) and total (PAK) PAK levels in uninfected cells and EPEC-infected cells treated with dimethyl sulfoxide (DMSO) (control) or the PAK inhibitor G5555, NVS PAK1-1, or IPA3. (C) Quantification of active PAK (arbitrary units [a.u.]) from the immunoblot in panel B, normalized for total PAK. Each bar represents the average of results from 3 separate experiments, and error bars represent SD. ***, *P* < 0.001 (one-way ANOVA followed by a *post hoc* Dunnett comparison) relative to the control. (D) Fluorescence microscopy images of pedestals formed by WT EPEC on Hap1 cells treated with DMSO (control) or the PAK inhibitor G5555, NVS-PAK1-1, or IPA3. Actin is stained with Alexa Fluor 488-phalloidin (green), and bacteria are stained with an anti-intimin antibody (red). Scale bar, 1 μm. (E) Immunoblot showing PAK1 and PAK2 knockdown efficiencies in ΔPAK2 and ΔPAK1 cells, respectively. (F) Fluorescence microscopy images showing the localization of green fluorescent protein (GFP)-tagged WT PAK1 and PAK^L107F^ (green), actin (Texas Red phalloidin [red]), and adherent EPEC (anti-intimin antibody [blue]). Individual channels are shown in gray scale, as indicated. Download FIG S2, PDF file, 0.6 MB. Copyright © 2019 Singh et al. 2019 Singh et al. This content is distributed under the terms of the Creative Commons Attribution 4.0 International license.

To determine the importance of EspG-mediated PAK activation, HAP1 cells were treated with various inhibitors of group I PAKs and then infected with WT EPEC. Immunoblotting confirmed that each inhibitor effectively blocked EPEC-driven PAK activation (Fig. S2B and C). Both the ability to form pedestals (Fig. S2D) and the ability to attach to cells (Fig. 2B) were greatly impeded (attachment decreased by up to 80%) in the presence of each of the PAK inhibitors. Both attachment (Fig. 2C) and pedestal morphology (Fig. 2D) were also greatly impaired in Hap1 knockout cells lacking either PAK1 (ΔPAK1) or PAK2 (ΔPAK2). The depletion of the other PAK isoform via small interfering RNA (siRNA) in the respective knockout cells (Fig. S2E) resulted in a further reduction in pedestal formation and attachment (Fig. 2C and D). These results imply that both PAK1 and PAK2 play a role in pedestal formation and attachment. Consistently with this, both WT PAK1 and the constitutively active mutant with an L107F mutation (PAK^L107F^) localize to actin pedestals (Fig. S2F).

### Arf6 is required for EspG to localize PAK to the plasma membrane.

**FIG 3:**
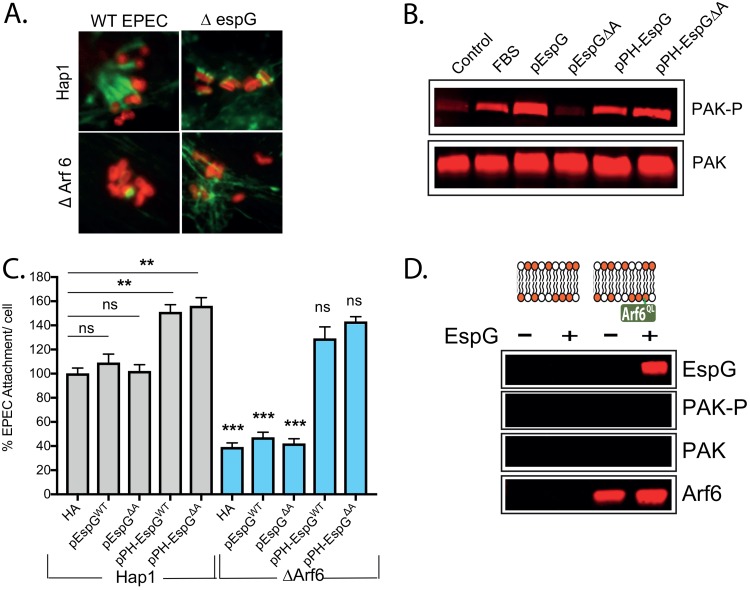
Arf6 is required for EspG’s localization to the plasma membrane. (A) Fluorescence microscopy images of actin pedestals formed on WT and ΔArf6 Hap1 cells by WT and Δ*espG* EPEC bacteria. Actin (green) is stained with Alexa Fluor 488-phalloidin, and bacteria (red) are stained with anti-intimin antibody. Scale bar, 1 μm. (B) Immunoblot representing levels of active PAK (PAK-P) in control Hap1 cells, cells treated with FBS, cells transfected with either WT (pEspG) or Arf-binding defective EspG (pEspGΔA), and the same cells fused to a PH domain (pPH-EspG and pPH-EspGΔA). (C) Attachment of WT EPEC to WT and ΔArf6 Hap1 cells transfected with a control empty plasmid (HA) or the different EspG variants listed for panel B. Values are relative to the attachment of WT EPEC to WT Hap1 cells (there are typically 6 to 7 WT EPEC bacteria per cell). Each bar represents an average of results from 3 separate experiments (300 to 500 cells for each experiment). Error bars represent SD. (D) Immunoblot showing recruitment of the indicated proteins from porcine brain extract by lipid bilayers alone or those loaded with Arf6^Q69L^, in the absence or presence of EspG. ***, *P* < 0.001; **, *P* < 0.01; ns, not significant (one-way ANOVA followed by a *post hoc* Tukey comparison) relative to the equivalent strain on WT Hap1 cells, except where indicated by a line.

Figure 1C and D show that, in addition to PAK, Arf GTPases are crucial for EspG-driven pedestal formation and attachment. Arf6 is the predominant Arf found at the plasma membrane and is the major family member bound by EspG during infection (20). WT and Arf6 knockout (ΔArf6) Hap1 cells were infected with either WT or Δ*espG* EPEC, and pedestal formation was assessed using fluorescence microscopy. Pedestals that formed on ΔArf6 cells were very small and similar morphologically to those formed by Δ*espG* EPEC (Fig. 3A). Consequently, WT EPEC attachment to ΔArf6 cells was also reduced (Fig. S3A). The addition of brefeldin A, an inhibitor of Golgi apparatus-localized Arfs (primarily Arf1 and -3), to ΔArf6 cells reduced the level of attachment slightly further, to the same level as that of Δ*espG* EPEC to WT cells. Attachment of Δ*espG* EPEC to ΔArf6 cells (or to ΔArf6 cells treated with brefeldin A) showed no additive defect compared to attachment to WT cells, suggesting that Arf6 and EspG function in the same pathway.

10.1128/mBio.01876-19.3 FIG S3 (A) Quantification of WT and Δ*espG* EPEC attachment to WT and ΔArf6 Hap1 cells, as well as ΔArf6 cells treated with the Arf GEF inhibitor brefeldin A (BFA). Values are relative to those of WT EPEC attachment to WT cells (typically 6 to 7 per cell). Each bar represents the average of results from 3 separate experiments (300 to 500 cells for each experiment). Error bars represent SD. ***, *P* < 0.001; ns, not significant (Student’s *t* test [indicated by lines] or one-way ANOVA followed by a *post hoc* Dunnett comparison [relative to WT Hap1 cells]). (B) Immunoblot of the level of active PAK (PAK-P) in WT and ΔArf6 Hap1 cells. Where indicated (+), cells were infected with WT EPEC and/or treated with brefeldin A. (C) Quantification (arbitrary units) of the level of active PAK in the EPEC-infected samples from panel B, normalized for total PAK. Each bar represents the average of results from 3 separate experiments, and error bars represent SD. ***, *P* < 0.001; **, *P* < 0.01 (one-way ANOVA followed by a *post hoc* Tukey comparison) relative to the same condition in WT Hap1 cells, except where indicated by a line. (D) Immunoblot of the amount of active PAK (PAK-P) in WT and ΔArf6 HAPs. Cells were not transfected (–) or transfected (+) with a plasmid encoding HA-tagged EspG, in the absence or presence of brefeldin A, as indicated. (E) Quantification (arbitrary units) of the level of active PAK in the transfected samples from panel E, normalized for total PAK. Each bar represents the average of results from 3 separate experiments, and error bars represent SD. ***, *P* < 0.001; **, *P* < 0.01 (one-way ANOVA followed by a *post hoc* Tukey comparison) relative to the same condition in WT Hap1 cells, except where indicated by line. (F) Localization of transfected Emerald-tagged EspG in WT and ΔArf6 Hap1 cells. Scale bar, 10 μm. (G) Attachment of Δ*espG* EPEC bacteria to Hap1 cells transfected with an empty plasmid (HA), WT EspG (pEspG^WT^), EspG unable to bind Arf (pEspG^ΔA^), or the same constructs fused to a PH domain (pPH-EspG^WT^ and pPH-EspG^ΔA^, respectively). Values are relative to those of WT EPEC attachment to control transfected (HA) WT Hap1 cells (typically 6 to 7 per cell). Each bar represents the average of results from 3 separate experiments (300 to 500 cells for each experiment). Error bars represent SD. ***, *P* < 0.001; **, *P* < 0.01 (one-way ANOVA followed by a *post hoc* Dunnett comparison), relative to WT EPEC attachment to control transfected (HA) WT Hap1 cells. (H) Immunoblot showing the level of recruitment of total (PAK) and active (PAK-P) PAK to empty bilayers (–) or those containing recombinant Cdc42^Q61L^ (+). Download FIG S3, PDF file, 0.1 MB. Copyright © 2019 Singh et al. 2019 Singh et al. This content is distributed under the terms of the Creative Commons Attribution 4.0 International license.

Surprisingly, PAK was still activated in ΔArf6 cells by either EPEC infection (Fig. S3B and C) or expression of hemagglutinin (HA)-tagged EspG (Fig. S3D and E). In both cases, PAK activation was, however, greatly diminished by the addition of brefeldin A. Arf6 is therefore important for pedestal formation and attachment yet not required for PAK activation. It is possible that Arf6 is required to localize EspG, and therefore active PAK, to the plasma membrane; in the absence of Arf6, EspG can bind to other Arfs and activate PAK at other membranes, such as the Golgi apparatus. Consistently with this hypothesis, while a fraction of transfected HA-tagged EspG localized to the plasma membrane in WT cells, this was absent in ΔArf6 cells (Fig. S3F, magnified insets).

To test whether Arf binding by EspG has a functional role beyond localization, we fused the pleckstrin homology (PH) domain from phospholipase Cδ1 to the N terminus of EspG and EspGΔA. This PH domain specifically binds phosphatidylinositol-4,5-bisphosphate (PIP2) and will therefore target these constructs to the plasma membrane. As previously shown (Fig. 2A), transfection of Hap1 cells with WT EspG triggers strong activation of PAK, whereas EspGΔA does not (Fig. 3B). However, fusing EspGΔA to a PH domain (PH-EspGΔA) restored the ability to activate PAK, suggesting that it is the ability to localize EspG to the membrane that is required for efficient PAK activation and not some other direct role for Arf binding. To test this hypothesis during infection, WT and ΔArf6 cells expressing the same constructs described in the legend of Fig. 3B were infected with WT EPEC. In WT Hap1 cells, both PH-EspG and PH-EspGΔA promoted attachment to levels higher than that of the control (Fig. 3C). More interestingly, while the attachment of EPEC overexpressing EspG or EspGΔA to ΔArf6 cells remained much less than its attachment to WT Hap1 cells, PH-EspG and PH-EspGΔA overexpression promoted enhanced attachment even to ΔArf6 cells (Fig. 3C). A similar experiment was also carried out using Δ*espG* EPEC to infect WT Hap cells, and here, HA-EspG, PH-EspG, and PH-EspGΔA, but not EspGΔA, were able to restore attachment (Fig. S3G).

It thus seems clear that (i) Arf is required to appropriately localize EspG during infection so that it can activate PAK at the necessary site within the cell and that (ii) Arf binding does not likely alter the activity of the protein. Consistently with these conclusions, it has previously been reported that EspG is sufficient to activate PAK in solution (21). We therefore attempted to reconstitute PAK recruitment to the membrane using lipid bilayer-coated silica microspheres. We anchored a constitutively active Arf mutant (Arf6^Q69L^) to the bilayers and tested whether they could recruit PAK from a cell-free porcine brain extract in the presence or absence of EspG. As expected, EspG associated with the bilayers only when Arf was present (Fig. 3D). However, unlike the control, Cdc42 (Fig. S3H), EspG failed to recruit any PAK. This suggests that an extra component is required for EspG to subvert PAK signaling.

### Hijacking of PAK by EspG requires Rho GTPases.

**FIG 4:**
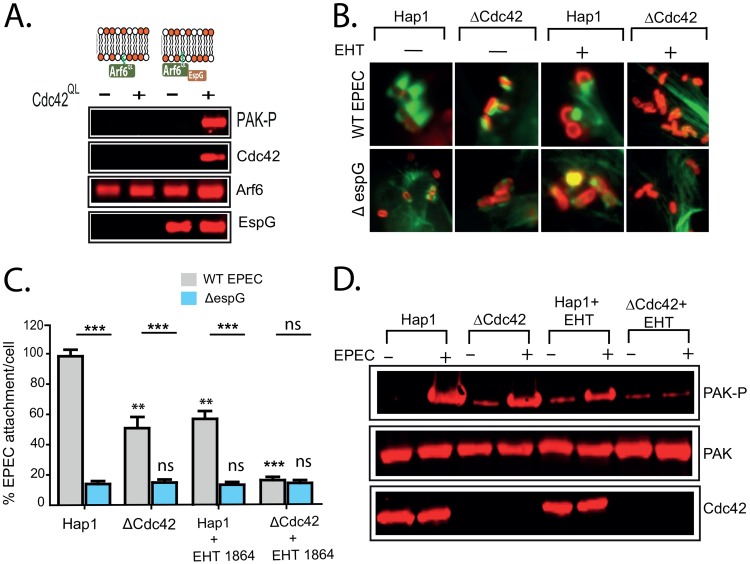
Small GTPases are required for EspG-driven recruitment of PAK. (A) Immunoblot showing recruitment of the indicated proteins from porcine brain extract by lipid bilayers loaded with either Arf6^Q69L^ alone or Arf6^Q69L^ together with EspG, with or without the addition of nonprenylated (soluble) Cdc42^Q61L^ to the extract. (B) Fluorescence microscopy images of pedestals formed by WT EPEC on WT and ΔCdc42 Hap1 cells in the presence and absence of the Rac1 inhibitor EHT1864 (EHT). Actin was stained with Alexa Fluor 488-phalloidin (green), and bacteria were stained with an anti-intimin antibody (red). Scale bar, 1 μm. (C) Attachment of WT and Δ*espG* mutant bacteria to WT and ΔCdc42 Hap1 cells, with and without EHT1864 treatment. Values are relative to WT EPEC attachment to WT Hap1 cells (there are typically 6 to 7 WT EPEC bacteria per cell). Each bar represents the average of results from 3 separate experiments (300 to 500 cells for each experiment). Error bars represent SD. (D) Immunoblot depicting levels of active PAK (PAK-P) in WT and ΔCdc42 Hap1 cells, with and without EHT1864 treatment and in the presence or absence of WT EPEC infection. ***, *P* < 0.001; **, *P* < 0.01; ns, not significant (one-way ANOVA followed by a *post hoc* Dunnett comparison) relative to the equivalent strain on WT Hap1 cells. Lines indicate significance between pairs of conditions determined by Student's *t* test.

Class I PAKs are activated and recruited to membranes by the Rho family GTPases Rac1 and Cdc42 (25). Addition of unprenylated Cdc42^Q61L^ efficiently activated PAK in brain extract (Fig. S4A). This preactivated PAK could not be recruited to lipid bilayers by Arf6^Q69L^ alone but was efficiently recruited when EspG was also present (Fig. 4A). Interestingly, the unprenylated (and therefore non-membrane-binding) Cdc42 was also found associated with the bilayers, suggesting that EspG recruits a PAK-GTPase complex to the membrane.

10.1128/mBio.01876-19.4 FIG S4 (A) Immunoblot showing levels of total (PAK) and active (p-PAK) PAK in porcine brain extract, in the absence (–) and presence (+) of recombinant Cdc42^Q61L^. (B) Immunoblot showing levels of total (PAK) and active (p-PAK) PAK in WT and Δcdc42 Hap1 cells transfected with HA-tagged EspG and treated with the Rac1 inhibitor EHT1864, as indicated. Download FIG S4, PDF file, 0.1 MB. Copyright © 2019 Singh et al. 2019 Singh et al. This content is distributed under the terms of the Creative Commons Attribution 4.0 International license.

Consistently with this requirement for Rho GTPases, pedestal formation (Fig. 4B) and attachment (Fig. 4C) by WT EPEC were significantly impaired in either Cdc42 knockout Hap1 cells (ΔCdc42) or WT Hap cells treated with the Rac1 inhibitor EHT1864 (Rac1 knockout Hap1 cells are severely compromised in adhesion to culture dishes and therefore could not be used for infection assays). When Rac1 was inhibited in the ΔCdc42 cells, both pedestal formation and attachment were reduced to the levels seen for Δ*espG* EPEC in WT Hap1 cells. Activation of PAK by either EPEC infection (Fig. 4D) or ectopic expression of EspG (Fig. S4B) was also reduced in both ΔCdc42 cells and WT cells treated with the Rac1 inhibitor and completely abolished when Rac1 was inhibited in ΔCdc42 cells.

**FIG 5:**
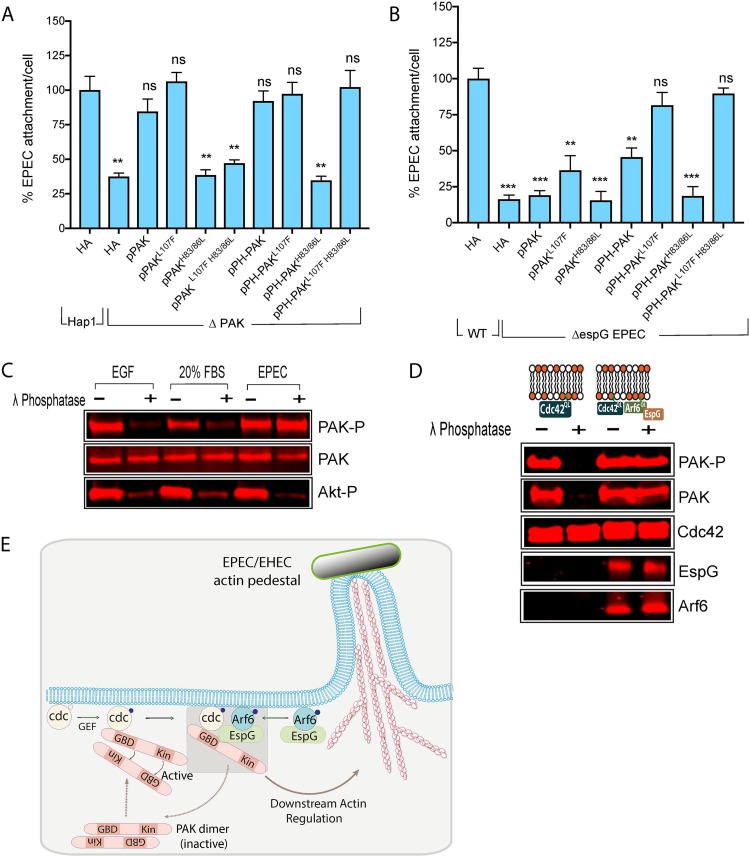
EspG promotes sustained PAK activation. (A) Quantification of WT EPEC attachment to ΔPAK1 Hap1 cells transfected with the indicated plasmids, encoding full-length PAK (pPAK), the constitutively active mutant (pPAK^L107F^), the GTPase binding-deficient mutant (pPAK^H83/86L^), or the combined mutant (pPAK^L107F H83/86L^), or the same derivatives fused to a pleckstrin homology domain (pPH-PAK, pPH-pPAK^L107F^, pPH-PAK^H83/86L^, and pPH-PAK^L107F H83/86L^). Values are relative to levels of attachment to control WT Hap1 cells transfected with an empty HA vector (there are typically 6 to 7 bacteria per cell). Each bar represents the average of results from 3 separate experiments (300 to 500 cells for each experiment). Error bars represent SD. (B) Attachment of Δ*espG* EPEC to WT Hap1 cells transfected with the same PAK constructs as in panel A. Values are relative to those of WT EPEC attachment to control transfected (HA) WT Hap1 cells (there are typically 6 to 7 bacteria per cell). Each bar represents the average of results from 3 separate experiments (300 to 500 cells for each experiment). Error bars represent SD. (C) Immunoblot depicting levels of active PAK (PAK-P) in Hap1 cells upon stimulation with EGF or 20% FBS or after infection with WT EPEC before (–) and after (+) subsequent treatment with λ phosphatase. (D) Immunoblot depicting recruitment of the indicated proteins to bilayers containing Cdc42^Q61L^ alone, Cdc42^Q61L^ plus Arf6^Q69L^ plus EspG when incubated in porcine brain extract, before (–) and after (+) treatment with λ phosphatase. (E) Model of the hijacking of PAK by EspG. See Discussion for a full description. ***, *P* < 0.001; **, *P* < 0.01; ns, not significant (one-way ANOVA followed by a *post hoc* Dunnett comparison) relative to the levels of attachment of WT EPEC to WT Hap1 cells.

To confirm the role of Rho GTPases during EPEC attachment, PAK1 knockout Hap cells (ΔPAK1) ectopically expressing various PAK mutants were infected with WT EPEC and attachment quantified (Fig. 5A). Expression of either WT PAK or a constitutively active, “open” form of PAK (PAK^L107F^) effectively restored the attachment of EPEC to ΔPAK1 cells to a level similar to that seen in WT cells. However, expression of a PAK mutant incapable of binding small GTPases (PAK^H83,86L^) failed to restore attachment. Surprisingly, a combined PAK^L107F H83,86L^ mutant, which although incapable of binding GTPases is constitutively open and active, was also unable to restore EPEC attachment. This suggests that the role of Rho GTPases is not simply activation of PAK.

As Rho GTPases are membrane localized, they may also play a role in concentrating PAK at the membrane prior to binding by EspG. Consistently with this, artificially localizing PAK^L107F H83,86L^ to the membrane by fusing it to a PH domain (PH-PAK^L107F H83,86L^) could restore EPEC attachment in ΔPAK1 cells (Fig. 5A). However, this was not the case for PH-PAK^H83,86L^ cells. This suggests that Rho GTPases have a role in both activating PAK and localizing it to the plasma membrane prior to being bound by EspG. To confirm this, we tested whether the various PAK derivatives could overcome the lack of Rho GTPase function in ΔCdc42 cells treated with the Rac1 inhibitor EHT1864 (Fig. S5A). Neither constitutive activation (PAK^L107F^) nor membrane localization of WT PAK (PH-PAK) could overcome the lack of Rho GTPase activity in these cells; however, EPEC attachment was restored by membrane-localized, constitutively active PH-PAK^L107F^ (Fig. S5A).

10.1128/mBio.01876-19.5 FIG S5 (A) Quantification of WT EPEC attachment to ΔCdc42 Hap cells pretreated with the Rac1 inhibitor EHT1864 and transfected with the indicated PAK constructs, as used in Fig. 5A. Values are relative to the level of attachment to WT Hap1 cells (typically 6 to 7 per cell). Each bar represents the average of results from 3 separate experiments (300 to 500 cells for each experiment). Error bars represent SD. **, *P* < 0.01; ns, not significant (one-way ANOVA followed by a *post hoc* Dunnett comparison), relative to levels of WT EPEC attachment to control transfected (HA) WT Hap1 cells. (B) Immunoblot showing levels of total (PAK) and active (p-PAK) PAK in Hap1 cells. Cells were untreated (control), treated with 10% FBS (FBS), treated with FBS followed by a 2-h incubation in serum-free medium (FBS + 2hr), infected with WT EPEC (EPEC), infected with WT EPEC followed by a 2-h incubation in serum-free medium (EPEC + 2hr) or infected with WT EPEC followed by a 2-h incubation in serum-free medium containing gentamycin (EPEC + 2hr Gent). (C) Immunoblot showing levels of total (PAK) and active (p-PAK) PAK, active Akt (p-Akt), and a loading control (tubulin) in Hap1 cells. Cells were incubated in serum-free medium (starved), treated with FBS (20% FBS), transfected with constitutively active Rac1 (Rac1^QL^), or transfected with HA-tagged EspG (EspG HA). Cell lysates were analyzed before (–) and after (+) treatment with λ phosphatase. Download FIG S5, PDF file, 0.1 MB. Copyright © 2019 Singh et al. 2019 Singh et al. This content is distributed under the terms of the Creative Commons Attribution 4.0 International license.

### EspG sustains active PAK for pedestal formation.

The data so far suggest that Rho GTPases both activate and localize PAK to the membrane for pedestal formation. What then is the function of EspG? To try to address this, we tested whether transfection of the various PAK derivatives used previously could overcome the defect in attachment when WT Hap1 cells are infected with Δ*espG* EPEC (Fig. 5B). Expression of constitutively active PAK (PAK^L107F^) increased the attachment of Δ*espG* EPEC only slightly, despite these cells expressing normal levels of Rho GTPases. However, attachment was restored to almost WT levels by fusing active PAK to the PH domain that specifically binds PIP2 (PH-PAK^L107F^). As PIP2 is enriched at sites of EPEC attachment, this suggests that although Rho GTPases are required to localize PAK to the membrane, recruitment to the specific site of pedestal formation requires EspG.

Surprisingly, although Rho GTPases were present, membrane-targeted WT PAK (PH-PAK) caused only a modest increase in the attachment of Δ*espG* EPEC (Fig. 5B). This suggests that although Rho GTPases are required for PAK activation, they are not sufficient for efficient EPEC attachment. Addition of fetal bovine serum (FBS) to resting cells causes an increase in PAK autophosphorylation, which is lost upon subsequent incubation of these cells in serum-free medium for 2 h (Fig. S5B). In contrast, infection of cells with EPEC leads to a large increase in PAK phosphorylation, which is not lost following subsequent incubation, even in the presence of antibiotics to kill the adherent bacteria (Fig. S5B). The loss of PAK autophosphorylation must be due to the actions of cellular phosphatases. We therefore tested whether EspG binding to PAK is able to block the actions of phosphatases. Addition of the nonspecific λ phosphatase to extracts from cells treated with either epidermal growth factor (EGF) or FBS was able to significantly reduce PAK phosphorylation (Fig. 5C). However, a similar treatment of extracts from EPEC-infected cells showed no such reduction. This protection from dephosphorylation was specific to PAK, as phosphorylation of AKT was abolished by λ phosphatase treatment. Similar protection was seen when PAK was activated by transfecting cells with EspG, whereas PAK activated by transfected Rac1 was still susceptible to dephosphorylation (Fig. S5C).

To confirm that this action was due to EspG, we reconstituted PAK activation *in vitro*. Purified Cdc42^Q61L^ anchored to lipid bilayers could efficiently recruit active PAK from porcine brain extract, and this recruited PAK was successfully dephosphorylated by exogenous λ phosphatase (Fig. 5D). However, when EspG was anchored via Arf6 to the bilayer alongside Cdc42, the recruited PAK could no longer be inactivated by the phosphatase. Together, these data show that EspG binding is able to protect PAK from phosphatase-mediated inactivation and, therefore, that it can sustain the PAK activation status.

## DISCUSSION

Collectively, the above data allow us to propose a model for the action of EspG during pedestal assembly (Fig. 5E). A proportion of the EspG injected by EPEC (and EHEC) localizes to the plasma membrane via binding to Arf GTPases, predominantly Arf6. The binding of PAK by cellular Rho GTPases both concentrates PAK in the membrane and exposes the Iα3 helix. This allows EspG to sequester active PAK to the site of bacterial attachment. In complex with EspG, PAK is protected from inactivation by phosphatases and plays a role in promoting the actin rearrangements necessary for efficient pedestal formation and attachment. Following assembly of this complex, the Rho GTPase may be released from PAK, allowing further PAK molecules to be activated. This may mean that only a small amount of active Rho GTPase is required to initiate low-level PAK signaling, which can then be effectively amplified by EspG. One of the effectors delivered by EPEC, mitogen-activated protein (MAP), is in fact a guanine nucleotide exchange factor (GEF) (activator) for Cdc42; however, deletion of MAP resulted in no significant difference in EPEC adhesion or PAK activation, either in the WT or in the ΔEspG strain (V. Singh, unpublished data). Identifying the pathway responsible for activating the required Rho GTPases therefore requires further study.

The precise role of EspG in pathogenesis has been controversial, perhaps unsurprisingly for a protein with multiple cellular targets. In a rabbit infection model, 10-fold-fewer bacteria were recovered from the colons of animals infected with ΔEspG EPEC than from those of animals infected with WT EPEC; however, no differences in symptoms, such as diarrhea, were observed (15). No infection defect was observed when the EspG homologue was deleted from Citrobacter rodentium, a model for EPEC infection (27). However, in competitive infections with WT bacteria, virtually no Δ*espG Citrobacter* organisms were recovered from coinfected mice (27). A separate study found that ∼100-fold-fewer bacteria were recovered from mice infected with Δ*espG Citrobacter* than from those infected with the WT 6 days postinfection, though there was little difference after 10 days (28). Collectively, these results suggest that there may be a role for EspG in the early stages of host colonization.

Although EspG has been shown previously to localize directly beneath adherent bacteria (28), previous studies using cultured cells have failed to demonstrate a defect in either attachment or pedestal formation for Δ*espG* EPEC (15, 29). This is in stark contrast to our findings, which clearly show that Δ*espG* EPEC and EHEC are significantly impaired in both pedestal formation and the ability to tightly adhere to target cells. Both phenotypes can be restored by complementation with a plasmid encoding EspG, confirming that these defects are EspG dependent. Our results show that Δ*espG* EPEC bacteria do form pedestals, but their formation is delayed compared to that of the WT (Fig. 1). It is possible that previous studies measuring pedestal number at late time points may have missed the phenotype, as at late times, the mutant bacteria have had time to “catch up.” However, our findings also show that the pedestals that are produced by Δ*espG* EPEC are much shorter and contain less actin than those produced by the WT; consequently, the bacteria adhere less strongly to cells (Fig. 1). This phenotype may have been missed previously due to differences in the precise cell lines, culture conditions, and infection protocols used (see below).

A second surprising finding was the requirement of Rho GTPases for EspG-dependent PAK recruitment and consequent pedestal formation. Previously, it has been reported that either expression of dominant negative Rho GTPase constructs or treatment of cells with a toxin that inhibits Rho GTPases has no effect on EPEC pedestal formation (30, 31). It is possible that the reason that previous studies have failed to find a role for EspG in pedestal formation may also be the reason that Rho GTPases have not been shown to be required; as the function of the Rho GTPase is to allow EspG to bind and recruit PAK, only under conditions where EspG is required would Rho GTPases also be required. Precisely what these conditions are remain to be determined; however, it is worth noting that there are multiple pathways in the cell which lead to PAK activation. It is tempting to speculate that EspG is not required if the level of active PAK in a cell is already above a certain threshold, which may be related to the precise cell line used or the conditions of culture/infection. Most of the experiments described here used Hap1 cells, due to the ready availability of numerous knockout clones. The Hap1 line encodes a breakpoint cluster region protein-Abelson kinase (BCR-ABL) fusion, which renders the tyrosine kinase Abl constitutively active. While it is possible that this influences the PAK signaling pathway, Δ*espG* EPEC bacteria were also defective in pedestal formation and attachment on multiple different cell lines lacking enhanced tyrosine kinase activity (Fig. S1A to C).

Previous studies have concluded that EspG is able to directly activate PAK in a Rho GTPase-independent manner, which seemingly contradicts our findings (21). However, the data from these previous studies actually support our results. All previous studies looking at the interaction between PAK and EspG have used fragments derived from PAK corresponding to the EspG binding site, rather than full-length PAK. Our results suggest that in cells, Rho GTPases are needed to bind to the inactive PAK homodimer in order to expose this binding site and allow EspG to bind. The only time that direct activation of PAK has been reported was when PAK immunoprecipitated from cells was used (21). At high concentrations *in vitro*, EspG may be able to bind to PAK; however, our results strongly suggest that this is not achieved in cells in the absence of cooperating Rho GTPases. It is uncertain whether the immunoprecipitated PAK used by Selyunin et al. (21) copurified with cellular GTPases or whether it was already partially active and/or autophosphorylated. Indeed, when the same group used a pseudoinactive kinase complex, composed of bacterially produced fragments corresponding to the kinase domain and the autoinhibitory domain (AID), EspG was unable to bind and induce kinase activity, whereas Cdc42 could (32). Collectively, these data are consistent with a model where Rho GTPase binding to PAK is required to expose the EspG binding site. Previous data (21) suggest that binding by EspG induces further conformational changes leading to enhanced kinase activity. Interestingly, it has been reported that following activation by a Rho GTPase, PAK2 can be “superactivated” by phosphorylation at tyrosine 135 (33). As this tyrosine is in the EspG binding site, it is tempting to speculate that in addition to stabilizing the active conformation of PAK (Fig. 5), binding by EspG may lead to an analogous superactivation, above the level induced by the GTPase alone.

It is not unprecedented for a microbial protein to specifically target preactivated PAK. The human immunodeficiency virus (HIV) encodes a protein called Nef (negative factor). Nef has been shown to specifically bind to the pool of already-active PAK within infected cells (34) and recruit it to specific membrane microdomains (35). As with EspG, mutants of Pak unable to bind Rho GTPases do not interact with Nef (36). The precise role of the interaction between Nef and PAK is uncertain, but by recruiting active PAK to the plasma membrane, the cytoskeletal regulator cofilin is phosphorylated and thus inactivated, thereby impairing T-cell receptor signaling (37).

Precisely how PAK contributes to pedestal formation remains to be determined. It has long been known that PAK influences cytoskeletal dynamics. Multiple cellular proteins have been identified as the substrates for PAK-mediated phosphorylation, several of which have obvious potential roles in controlling the cytoskeletal rearrangements that drive pedestal production (25). For example, PAK1-mediated phosphorylation increases the activity of LIM domain kinase 1 (LIMK1), which in turn phosphorylates and inhibits cofilin, an actin-severing protein (38). PAK1 also phosphorylates and activates cortactin, leading to Arp2/3-dependent actin polymerization (39). Arp2/3 itself can also be regulated by PAK, via phosphorylation of the ArpC1b subunit (40). It is important to note that in addition to functioning as a kinase, PAK acts as a scaffolding protein (41). The same conformational changes that relieve autoinhibition of the kinase domain also expose binding sites for various other proteins, especially via the N-terminal polyproline domains. Thus, activation of PAK triggers the recruitment of specific proteins into signaling complexes at the membrane. One example of this is the recruitment of the phosphatase PP2A by PAK1, which regulates the phosphorylation status of myriad proteins, including cytoskeletal regulators, such as ezrin/radixin/moesin (ERM) proteins (42). Further study is required to identify precisely which pathways downstream of PAK contribute to actin pedestal dynamics. Nevertheless, we have identified PAK as a key regulator of the cytoskeletal changes underlying pedestal formation and consequent bacterial adhesion to host cells and provide new insights into the molecular mechanism by which EspG manipulates the PAK signaling axis.

## MATERIALS AND METHODS

### Bacterial strains.

EPEC E2348/69 and EHEC EDL933 (Shiga toxin-deficient TUV93-0 derivative) strains were used. Isogenic mutant EPEC Δ*espG1* Δ*espG2* and EHEC Δ*espG* were generous gifts from Feng Shao and Ken Campellone, respectively.

### Plasmids.

pTrcEspG, pTrcEspGΔR, pTrcEspGΔP, and pTrcEspGΔAP were described previously (26). HA-EspG was generated using Gateway methodology (Invitrogen). HA-PAK1, HA-PAK1^L107F^, and HA-PAK1^H83,86L^ were generated by subcloning them from WT pCMV6M-Pak1, pCMV6M-Pak1 L107F, and pCMV6M-Pak1 H83L H86L, which were a gift from Jonathan Chernoff (43, 44) (Addgene plasmids 12209 [http://www.addgene.org/12209/; RRID:Addgene_12209], 12212 [http://www.addgene.org/12212/; RRID:Addgene_12212], and 12211 [http://www.addgene.org/12211/; RRID:Addgene_12211], respectively).

pTrc EspGΔA (E392R) was generated by site-directed mutagenesis (SDM) of pTrcEspG. HA-PAK1^L107F H83,86L^ was generated by SDM using HA-PAK1^H83,86L^ as the template. DNA encoding PH domain fusions to EspG, PAK1, and the various PAK1 mutants was synthesized (IDT Technologies) and cloned into expression vectors using the Gateway system (Invitrogen).

### Antibodies.

Antibodies were supplied by the following: Cell Signaling Technology (phospho-PAK1 [Ser144]/PAK2 [Ser141], catalog no. 2606; PAK1, 2602; PAK2, 2608; phospho-Akt [Ser473], 9271), Abcam (Rac1, ab33186; Arf6, ab81650; tubulin, ab7291), Sigma (actin, A2066), BD Biosciences (Cdc42, 610929), and Qiagen (His tag, 34660). Rabbit anti-intimin was raised against full-length recombinant intimin by Diagnostics Scotland.

### Mammalian cell culture.

WT Hap1 (C631) and verified-knockout ΔArf6 (HZGHC003403c006), ΔCdc42 (HZGHC003404c010), ΔPak1 (HZGHC000160c012), and ΔPak2 (HZGHC000053c001) lines were purchased from Horizon Discovery. Hap1 cells were maintained in Iscove’s modified Dulbecco’s medium (IMDM) supplemented with 10% FBS and 100 U/ml penicillin-streptomycin. HeLa cells, MEFs, and Caco-2 cells were maintained in Dulbecco’s modified Eagle’s medium (DMEM) with 10% FBS and 100 U/ml penicillin-streptomycin. Where indicated, cells were preincubated for 60 min with 10 μM G5555, NVS-PAK1-1 (all Tocris), IPA-3 (Sigma), EHT1864 (Merck), or 1 μM brefeldin A (Sigma). Where indicated, cells were transfected using the Neon system (Invitrogen) according to the manufacturer’s instructions.

Oligonucleotides (Qiagen) used to knock down the expression of PAK1 (HS_PAK1_1, AAGATTAACTTGGATCTTCTA; HS_PAK1_2, ACCCTAAACCATGGTTCTAAA) and PAK2 (HS_PAK2_1, ACGAGTAATTGTGAAGCATAA; Hs_PAK2_4, TGCAGTAGTATAAATCATGAA) were transfected using Oligofectamine (Life Technologies) according to the manufacturer’s instructions.

### Attachment assay and pedestal quantification.

Cells were infected as described previously (45). Cells were washed three times with phosphate-buffered saline (PBS), fixed, and stained with Alexa Fluor 488-phalloidin (Lifetech) to visualize actin and with an anti-intimin antibody to visualize the bacteria, and the number of actin pedestals per cell was counted using fluorescence microscopy. For adhesion assays, cells were washed twice with PBS and then twice briefly with 200 mM glycine (pH 2), followed by a further two washes with PBS. Cells were fixed and stained as described above, and the number of adherent bacteria was counted using microscopy.

### *In vitro* pulldown assays.

Pulldown assays were performed as described previously (26). Briefly, silica microspheres (Bangs Laboratories) were coated with a bilayer composed of an equal molar ratio of phosphatidylcholine and phosphatidylserine (Avanti Polar Lipids). The proteins indicated in Results were anchored to these bilayers prior to incubation in cell-free porcine brain extract (46). Following incubation for 15 min, bilayers were washed extensively and the associated proteins analyzed by SDS-PAGE. Recombinant EspG, Arf6^Q69L^, and Cdc42^Q61L^ were purified and lipid modified as described previously (26).

### PAK activation assays.

Cells were cultured in serum-free IMDM overnight, infected or treated with appropriate drugs as indicated in the figures, and then washed twice with PBS before being scraped and resuspended in SDS-urea. Alternatively, cells were lysed using radioimmunoprecipitation assay (RIPA) buffer (Sigma) prior to treatment with lambda phosphatase (Sigma) according to the manufacturer’s instructions. Samples were analyzed by SDS-PAGE and immunoblotting using appropriate antibodies. Immunoblots are representative of at least three separate repeats. Bands were visualized using a LI-COR Odyssey Fc imaging system, and band intensities were quantified using the LI-COR Image Studio software.
