# Supplementary figures and images for: Pathogenic Escherichia coli Hijacks GTPase-Activated p21-Activated Kinase for Actin Pedestal Formation
Source: mBio. 2019 Aug 20;10(4):e01876-19. doi: 10.1128/mBio.01876-19 (PMC6703428; doi:10.1128/mBio.01876-19)

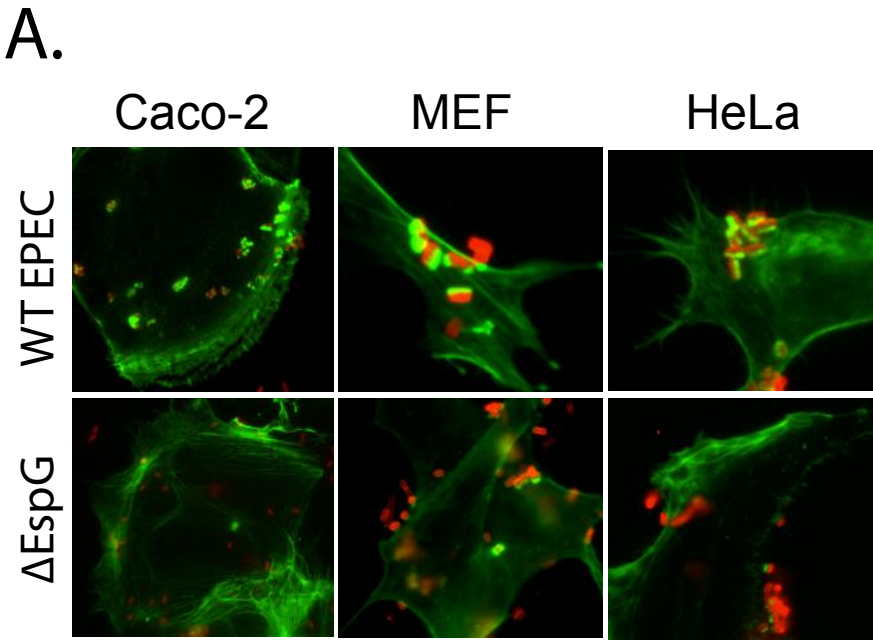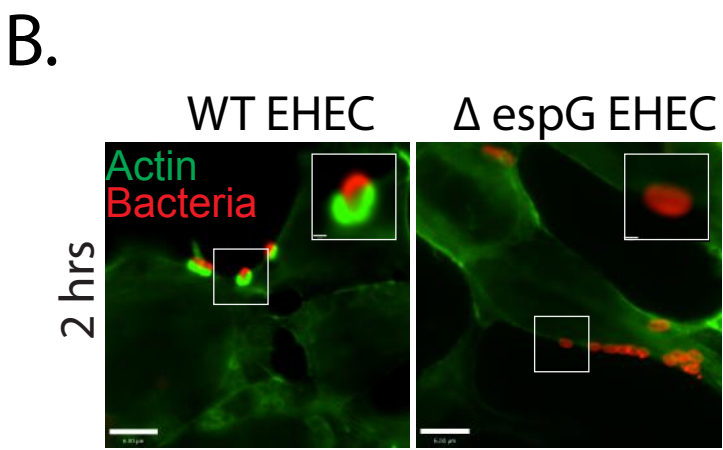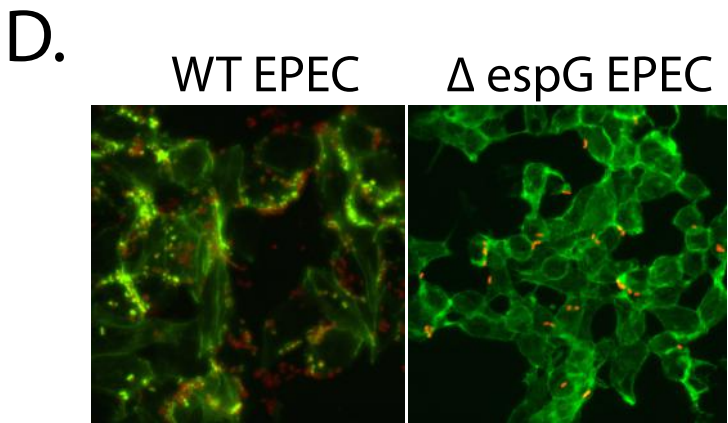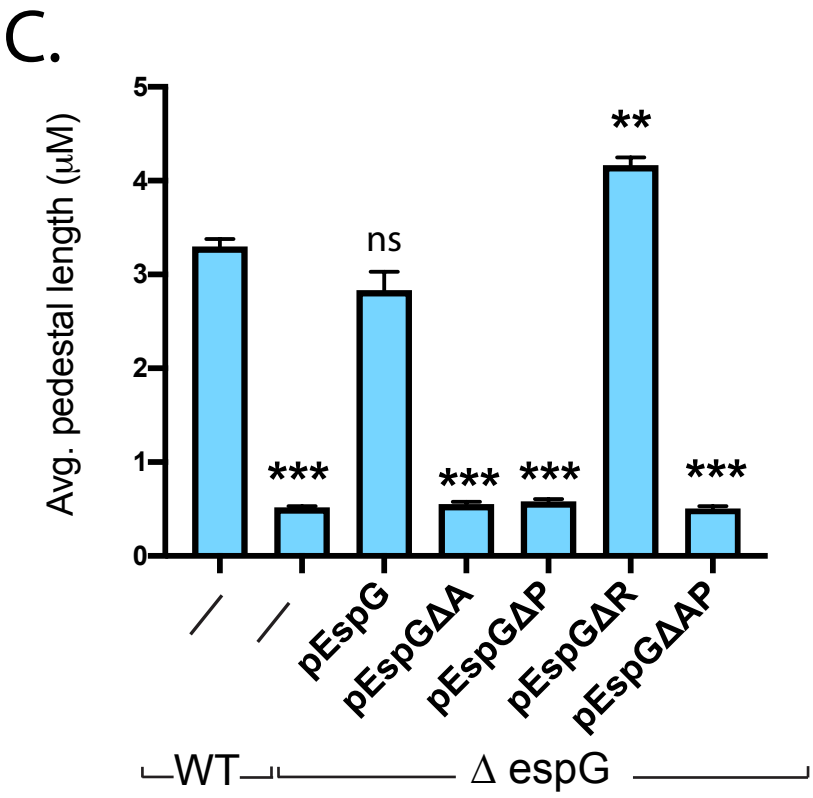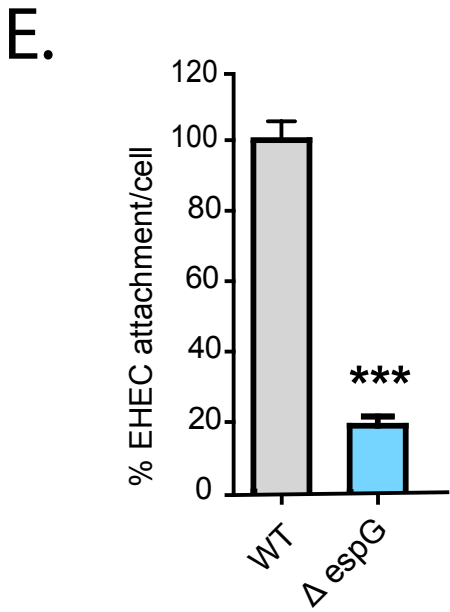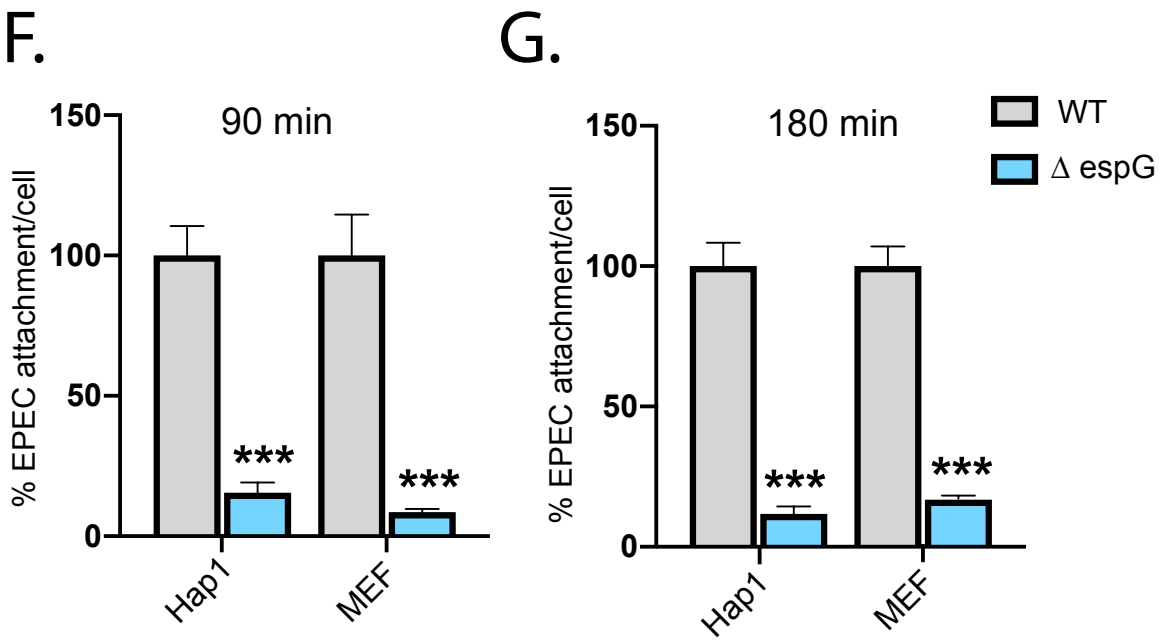

Supplement: FIG S1 [file mBio.01876-19-sf001.pdf]

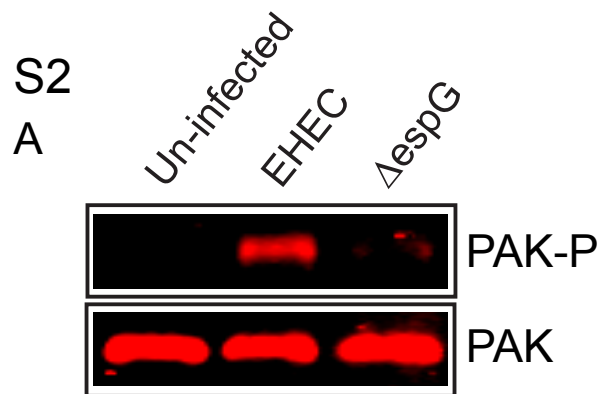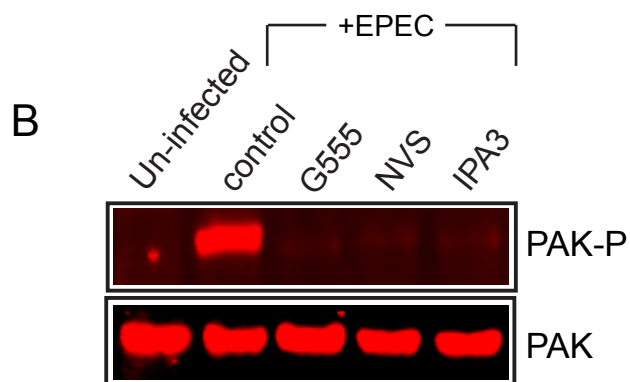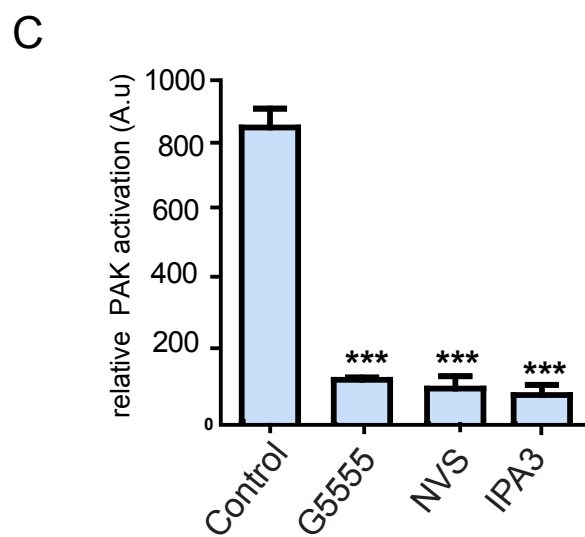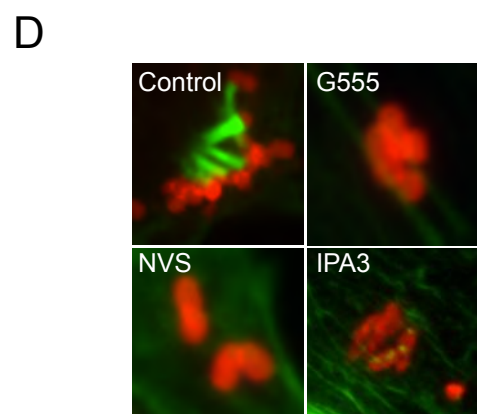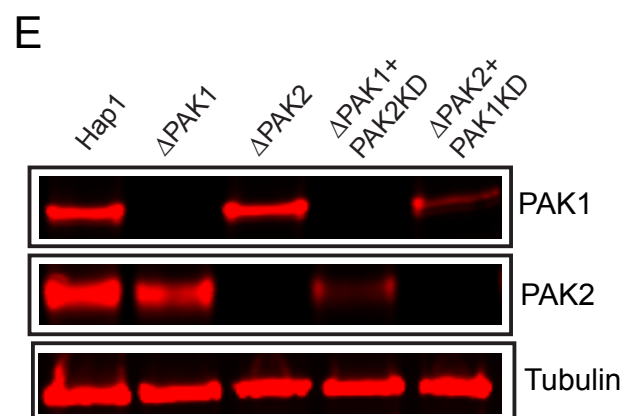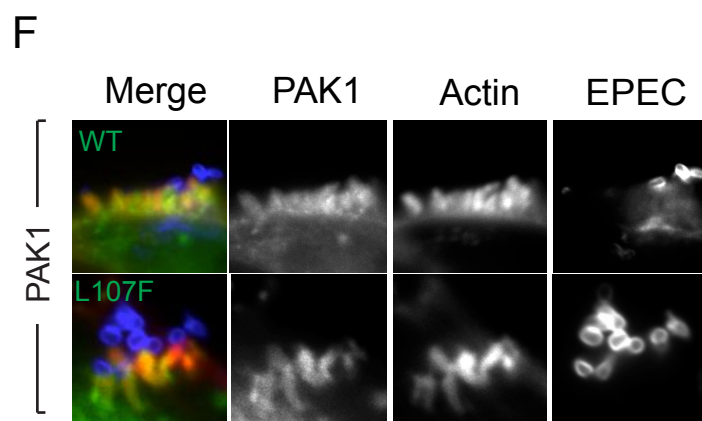

Supplement: FIG S2 [file mBio.01876-19-sf002.pdf]

S3.

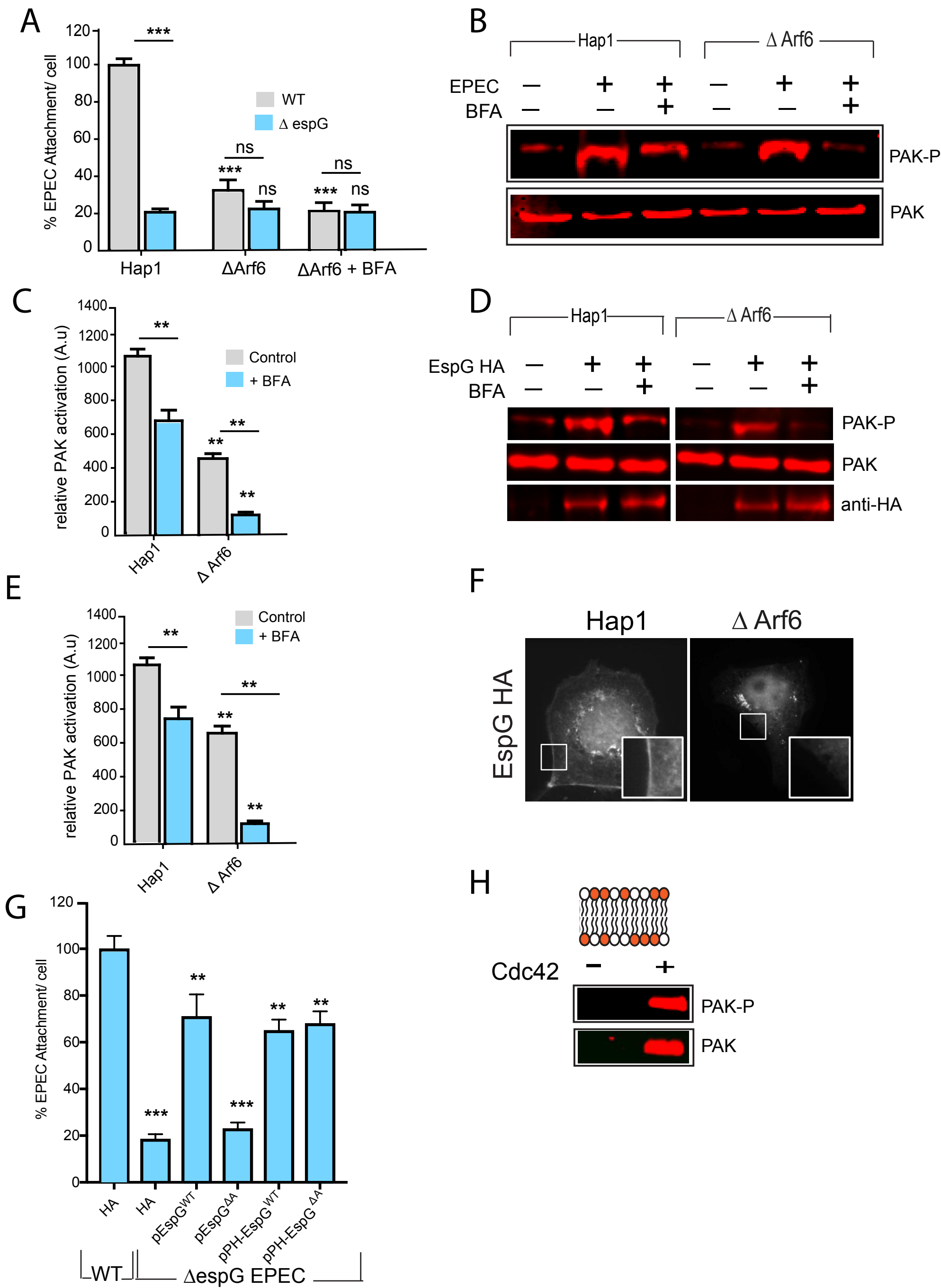

Supplement: FIG S3 [file mBio.01876-19-sf003.pdf]

A

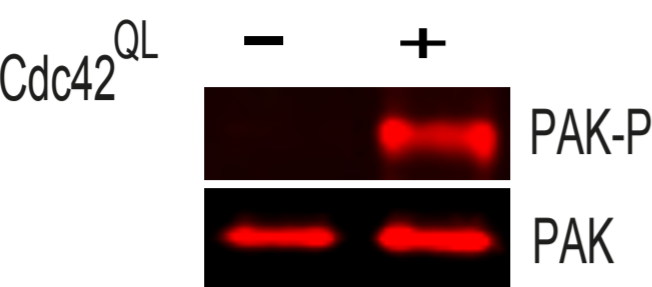

B

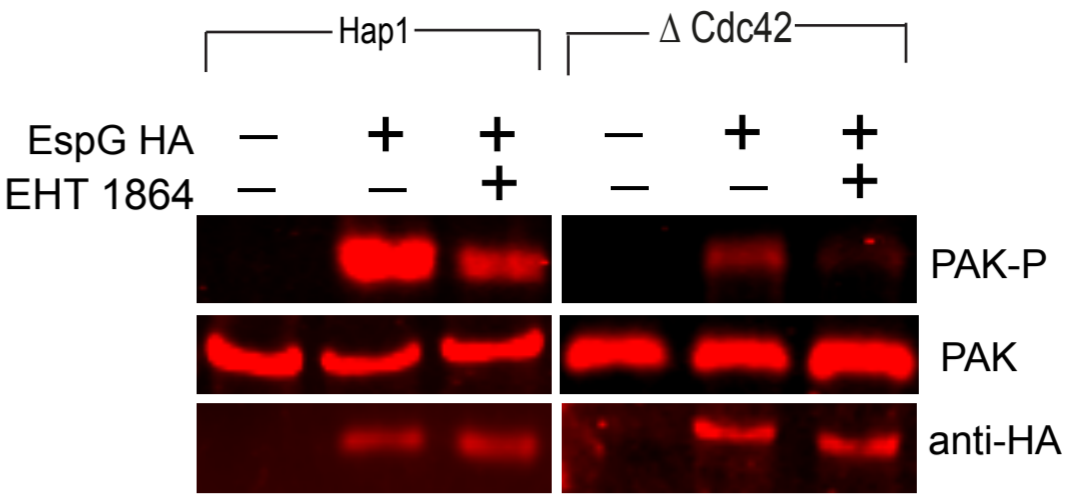

Supplement: FIG S4 [file mBio.01876-19-sf004.pdf]

S5

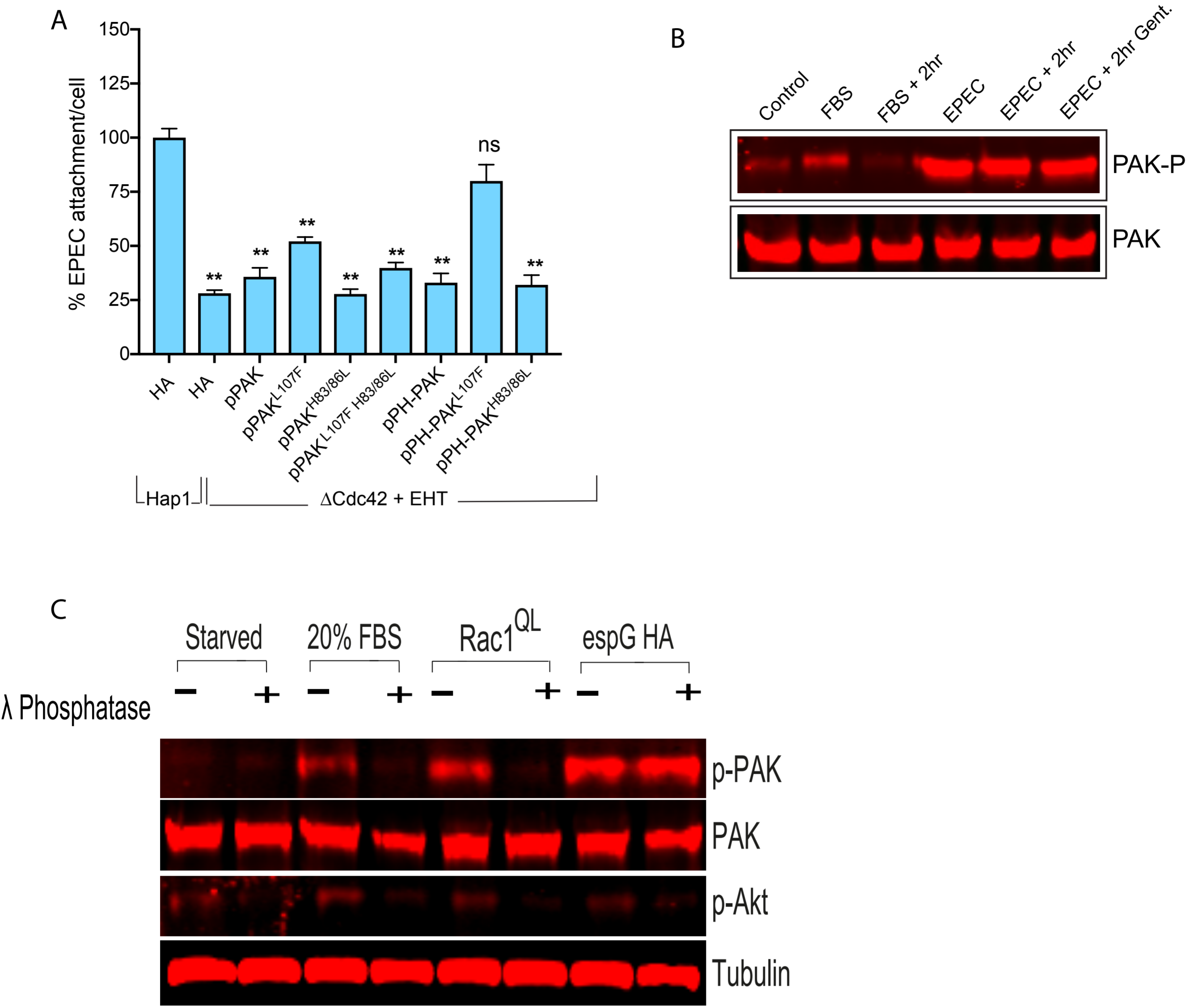

Supplement: FIG S5 [file mBio.01876-19-sf005.pdf]
